# Seroprevalence of *Toxoplasma gondii *infection in dairy goats in Shaanxi Province, Northwestern China

**DOI:** 10.1186/1756-3305-4-47

**Published:** 2011-04-01

**Authors:** Guang-Hui Zhao, Miao-Tao Zhang, Li-Hui Lei, Chuan-Chuan Shang, Duo-Yao Cao, Ting-Ting Tian, Jie Li, Jun-Yan Xu, Yun-liang Yao, De-Kun Chen, Xing-Quan Zhu

**Affiliations:** 1 College of Veterinary Medicine, Northwest A & F University, Yangling, Shaanxi Province 712100, PR China; 2 State Key Laboratory of Veterinary Etiological Biology, Key Laboratory of Veterinary Parasitology of Gansu Province, Lanzhou Veterinary Research Institute, CAAS, Lanzhou, Gansu Province 730046, PR China; 3 Department of Animal Husbandry and Veterinary Medicine, Beijing Vocational College of Agriculture, Beijing 102442, PR China; 4 College of Animal Science and Veterinary Medicine, Heilongjiang Bayi Agricultural University, Daqing, Heilongjiang Province 163319, PR China; 5 College of Animal Science and Technology, Yunnan Agricultural University, Kunming, Yunnan Province 650201, PR China

## Abstract

**Background:**

*Toxoplasma gondii *is an important zoonotic pathogen causing significant human and animal health problems. Infection in dairy goats not only results in significant reproductive losses, but also represents an important source of human infection due to consumption of infected meat and milk. In the present study we report for the first time seroprevalence of *T. gondii *infection in Guanzhong and Saanen dairy goats in Shaanxi province, Northwestern China.

**Results:**

Sera from 751 dairy goats from 9 farms in 6 counties were examined for *T. gondii *antibodies with an indirect haemagglutination (IHA) test. Antibodies to *T. gondii *were detected in 106 (14.1%) serum samples, with antibody titres ranging from 1:64 to 1:1024. Seropositive goats were found in all 9 farms and seroprevalences in Guanzhong (16.3%, 75/461) and Saanen (10.7%, 31/290) dairy goats were not statistically significantly different. All the factors (sex, age and location) reported in the present study affected prevalence of infection, and seroprevalence increased with age, suggesting postnatal acquisition of *T. gondii *infection.

**Conclusions:**

The results of the present survey indicate that infection by *T. gondii *is widely prevalent in dairy goats in Shaanxi province, Northwestern China, and this has implications for prevention and control of toxoplasmosis in this province.

## Background

*Toxoplasma gondii *can infect nearly all the warm-blooded animals, including mammals and birds throughout the world [1-4]. Infection in dairy goats not only results in significant reproductive losses, but also represents an important source of human infection due to consumption of infected meat and milk constituting zoonotic transmission [3,5-8]. The seroprevalence of *T. gondii *in goats has been surveyed in many countries, and these worldwide reports were recently summarized [3]. Viable *T. gondii *was isolated from goats killed for human consumption [9,10].

The People's Republic of China (PRC) is one of the largest producers of dairy goats in the world, and Shaanxi Province is the major dairy goat producer in the PRC. Table 1 summarizes reports of *T. gondii *infection in goats from the PRC because these papers were published in the Chinese language in local journals and are not easily accessible to foreign scholars. In the present study we report seroprevalence of *T. gondii *infection in dairy goats in Shaanxi province, Northwestern China for the first time.

**Table 1:** Prevalence of *Toxoplasma gondii *infection in goats in People's Republic of China (PRC)

Usage	Provinces/cities	No. tested	Positive (%)	Serologic test^a^	Cut-off value	Time tested (year)	References
Meat	Gansu (Tianzhu)	1028	26.1	IHA	1:64	1995	[11]
Meat	Yunnan (Honghe)	3925	30.8	IHA	1:64	Unknown	[12]
Meat	Beijing	230	39.1	PA	+^b^	Unknown	[13]
Meat	Qinghai (Datong)	1128	24.9	IHA	1:64	Unknown	[14]

^a ^IHA: indirect hemagglutination test, PA: Plate agglutination.

^b ^Occurrence of particle agglutination.

## Methods

### Study animals

Blood samples were obtained from 751 dairy goats in September and October, 2010 from 9 randomly selected farms in 6 counties/district in Shaanxi Province. Details of management, source and breeds of goats, and other characteristics are summarized in Table 2. Animals were farmed in extensive production systems for meat and milk and were generally kept in small herds of 20-100 animals. Natural breeding was the sole means of reproduction and goats from outside breeding stocks was rarely purchased. Goats were fed in-house with no grazing. In local practice, both Guanzhong and Saanen dairy goats were crossed with Saanen male goats, therefore, our study included only male goats for the Saanen breed. Of the 9 sampled farms, only one farm (Qianyang county) was for breeding goats.

**Table 2:** Factors associated with seroprevalence of *Toxoplasma gondii *infection in dairy goats in Shaanxi Province, Northwestern China

Factor	Category	No. examined	No. positive (%)	Exp (95% CI)	*P*
Breed	Saanen dairy goat	290	31 (10.7)	--	
	Guanzhong dairy goat	461	75 (16.3)	2.233 (0.406, 12.269)	0.356
Sex					
	Male	70	11 (15.7)	0.259 (0.080, 0.833)	0.023
	Female	681	95 (14.0)	--	
Age					
	<1 year	175	17 (9.7)	0.363 (0.189, 0.696)	0.696
	1-2 year	67	6 (9.0)	0.612 (0.227, 1.646)	0.331
	>2 year	509	83 (16.3)	--	0.126
Location (farms)					
	Zhuangli town, Fuping county	89	5 (5.6)	--	
	Wangliao town, Fuping county	114	42 (36.8)	13.031 (4.830, 35.158)	0.000
	Dongshangguan, Fuping county	126	19 (15.1)	3.105 (1.108, 8.698)	0.483
	Mizi town, Fuping county	157	12 (7.6)	1.474 (0.499, 4.349)	0.585
	Yangling district	123	14 (11.4)	5.479 (0.938, 31.997)	0.059
	Qianyang county	39	2 (5.1)	1.584 (0.195, 12.834)	0.667
	Fengxiang county	31	5 (16.1)	9.030 (1.093, 74.597)	0.041
	Baishui county	29	2 (6.9)	2.823 (0.265, 30.105)	0.390
	Chunhua county	43	5 (11.6)	5.213 (0.638, 42.576)	0.123
	Total	751	106 (14.1)		

### Blood sampling and serological examination

Approximately 3 ml of blood were obtained via a jugular vein, centrifuged at 2000 *g *for 5 min and stored at -20°C. Antibodies to *T. gondii *were determined in sera using an indirect hemagglutination antibody (IHA) test with a commercially available kit (Lanzhou Veterinary Research Institute, Chinese Academy of Agricultural Sciences, Lanzhou, Gansu Province, China) according to the manufacturer's instructions. In brief, sera were added to 96 well V bottomed polystyrene plates, and diluted in a four-fold series from 1:4 to 1:2048. The plates were shaken for 2 min and then incubated at 37°C for 2 h without shaking. The test was considered positive when a layer of agglutinated erythrocytes was formed in wells at dilutions of 1:64 or higher, and positive and negative controls were included in each test.

### Statistical analysis

Differences in seroprevalence of infected goats between the two breeds and among associated factors were analyzed using the binary logistic regression in SPSS for Windows (Release 17.0 standard version, SPSS Inc., Chicago, IL, USA), 95% confidence intervals (CI) are given. The Differences between levels within factors and interactions were considered to be statistically significant and highly significant when *P *< 0.05 and *P *< 0.01, respectively.

## Results and discussion

Antibodies to *T. gondii *were found in 106 (14.1%) of 751 goats with titres of 1:64 in 79 dairy goats, 1:256 in 16 dairy goats and 1:1024 in 11 dairy goats. Both Saanen and Guanzhong dairy goats were positive for *T. gondii *antibody, with higher prevalence in Guanzhong dairy goats than in Saanen dairy goats. The binary logistic regression showed that all the factors (sex, age and location) reported in the present study affected prevalence of infection. The seroprevalence in male goats (15.7%) was higher than that in females (14.0%), and the difference was statistically significant (Exp = 0.259, CI = 0.080-0.833, *P *= 0.023) (Table 2). Seroprevalence in goats increased progressively with age, and prevalence in older goats (>2-year-old) was higher than that in animals below 2-year-old. Seroprevalence at the individual farms ranged from 5.1% to 36.8% and seropositive goats were found in all 9 farms (Table 2).

In the present study, the overall seroprevalence was 14.1%, which was far less than other reports from the PRC (Table 1). The difference could be associated with ecological conditions, life styles of inhabitants, climates, husbandry practice and the numbers of cats and rodents present. The present study showed that the breeding dairy goats had the lowest prevalence (Table 2), possibly because breeding goats have better welfare and relatively less chance to come into contact with cats and rodents that play a significant role in the transmission of *T. gondii*. The Guanzhong dairy goat is a unique goat breed in Shaanxi province. The prevalence in Guanzhong dairy goats was different among individual farms, ranging from 5.6% to 36.8%, which is slightly higher than that in Saanen dairy goats. The differences may be attributed to breed differences in susceptibility to *T. gondii*. The present study showed that older dairy goats (>2-year-old) were more likely to be seropositive than goats under 2-year-old, which provided further evidence for the increased risk of *T. gondii *infection with acquisition of age through ingestion of infective oocysts from the environment.

## Conclusions

The results of the present survey indicated that infection of dairy goats with *T. gondii *is widespread in Shaanxi Province, China, which is of public health concern and has implications for prevention and control of toxoplamosis in this province. Therefore, integrated control strategies and measures are recommended to prevent and control *T. gondii *infection in dairy goats.

## Competing interests

The authors declare that they have no competing interests.

## Authors' contributions

GHZ, MTZ, LHL and CCS performed the study, managed, analyzed, and interpreted the data, and prepared the manuscript; DYC, TTT, JL and YLY facilitated and assisted the study implementation; MTZ and LHL contributed to the revision of the manuscript; XQZ and DKC designed the study, supervised the study implementation and revised the manuscript. All authors read and approved the final manuscript.

## Authors' information

^1^College of Veterinary Medicine, Northwest A & F University, Yangling, Shaanxi Province 712100, PR China. ^2^State Key Laboratory of Veterinary Etiological Biology, Key Laboratory of Veterinary Parasitology of Gansu Province, Lanzhou Veterinary Research Institute, CAAS, Lanzhou, Gansu Province 730046, PR China. ^3^Department of Animal Husbandry and Veterinary Medicine, Beijing Vocational College of Agriculture, Beijing 102442, PR China. ^4^College of Animal Science and Veterinary Medicine, Heilongjiang Bayi Agricultural University, Daqing, Heilongjiang Province 163319, PR China. ^5^College of Animal Science and Technology, Yunnan Agricultural University, Kunming, Yunnan Province 650201, PR China.
